# Organizational characteristics conducive to the implementation of health programs among Latino churches

**DOI:** 10.1186/s43058-020-00052-2

**Published:** 2020-07-06

**Authors:** Jennifer D. Allen, Rachel C. Shelton, Lindsay Kephart, Lina Jandorf, Sara C. Folta, Cheryl L. Knott

**Affiliations:** 1 Department of Community Health, Tufts University, 574 Boston Avenue, Medford, MA 02155 USA; 2 Department of Socio-medical Sciences, Columbia University Mailman School of Public Health, New York, NY 10032 USA; 3 Massachusetts Department of Public Health, 250 Washington Street, Boston, MA 02108 USA; 4 Department of Population Health Sciences and Policy, Icahn School of Medicine at Mount Sinai, One Gustave Levy Place, New York, NY 10029 USA; 5 Friedman School of Nutrition Science and Policy, Tufts University, 150 Harrison Ave., Boston, MA 02111 USA; 6 Department of Behavioral and Community Health, University of Maryland School of Public Health, 1234 W SPH Building, College Park, MD 20742 USA

**Keywords:** Implementation science, Faith-based organizations, Organizational readiness, Evidence-based interventions, Latinos, Cancer screening

## Abstract

**Background:**

Faith-based organizations (FBOs) can be effective partners in the implementation of health interventions to reach underserved audiences. However, little is known about the capacity they have or need to engage in these efforts. We examined inner-setting organizational characteristics hypothesized to be important for program implementation by the Consolidated Framework for Implementation Research (CFIR).

**Methods:**

This cross-sectional study involved 34 churches with predominantly Latino congregations in Massachusetts. FBO leaders completed a survey assessing inner-setting CFIR organizational characteristics, including organizational readiness, implementation climate, organizational culture, and innovation “fit” with organizational mission.

**Results:**

There was limited variability in CFIR organizational characteristics, with scores on a scale from 1 to 5 skewed toward higher values, ranging from 3.27 (SD 0.94) for implementation climate to 4.58 (SD 0.54). Twenty-one percent of the FBOs had offered health programs in the prior year.

**Conclusions:**

FBOs had high scores on most of the organizational factors hypothesized to be important for the implementation of health programs, although relatively few FBOs offered them. While this suggests that FBOs have favorable characteristics for health programming, prospective studies are needed to understand relative salience of inner-setting organizational characteristics versus factors external to the organization (e.g., policies, incentives), as well as the potential direction of relationships between internal organizational characteristics and health program offerings.

**Trial registration:**

Clinical trials identifier number NCT01740219 (clinicaltrials.gov)

Contributions to the literature

- FBOs can be instrumental in reaching underserved audiences, but little is known about the resources or capacity they have or need to offer health programs
- This is one of few studies to examine organizational factors of FBOs hypothesized to be important in the implementation of programs as hypothesized by the Consolidated Framework for Implementation Research (CFIR)
- FBOs in the sample had high scores on all measures of CFIR organizational characteristics thought to be important for program implementation, although relatively few FBOs had implemented health programs in the prior year
- Additional research is needed to understand prospective relationships between internal organizational characteristics and external factors that impact health program offerings.

## Background

Faith-based organizations (FBOs) can be valuable partners in the implementation of health promotion programs, particularly among communities that experience health inequities, structural barriers to accessing healthcare, and have high levels of mistrust of the healthcare system [1]. Over the past two decades, there has been a proliferation of intervention studies based in FBOs. Overall, empirical evidence supports that FBOs can be an important setting for and partner in the delivery of effective health interventions to address a wide variety of public health goals among diverse audiences [1–3].

With a solid body of evidence demonstrating that health promotion programs offered in FBOs can be effective, there is now a need to assess the potential for dissemination of interventions on a broader scale among FBOs and to a wider audience. This will require an understanding of the diverse “real world” contexts in which interventions are to be implemented. Organizational factors such as structural characteristics (e.g., size), as well as internal dynamics and processes (e.g., culture), have been found to be key factors in the successful adoption and implementation of programs in worksites [4], healthcare facilities [5], and schools [6]. Several studies have demonstrated that structural characteristics of FBOs (i.e., congregation size, number of personnel, existing infrastructure) are associated with higher levels of health programming [2, 7, 8]. However, to date, little attention has been given to other internal organizational characteristics or processes that may be critical to understanding the potential for dissemination efforts in FBOs.

The goal of this descriptive study was to assess the organizational factors specified by the Consolidated Framework for Implementation Framework (CFIR) to be associated with the implementation of new programs, policies, or practices among Latino-serving FBOs. We focus on Latino FBOs given health disparities experienced by Latinos and the high percentage that report church membership [9]. The vast majority of prior studies have been conducted in African American FBOs which may differ from Latino FBOs in terms of denomination, size, time since establishment, and available resources [10, 11]. As such, this study can contribute to understanding FBOs that identify as serving predominantly Latino congregations.

## Method

### Sample and setting

FBOs included in this study were located in Massachusetts, offered Spanish language religious services, and reported serving a predominantly Latino congregation. We included all denominations except for Catholic churches, since our prior research has focused extensively on the implementation of innovations among Catholic FBOs and there is now a need to study other denominations, as results from one may not be generalizable to others. Note that in this paper, we use the terms “FBOs” and “churches” interchangeably. Organizations were identified through the Worldwide Web (web) using search terms [“church” or “faith-based organization” AND “Latino” or “Hispanic” AND “Massachusetts”], as well as a review of listings in White Pages.

We mailed study materials to pastors in the identified churches, which included a project brochure outlining the study’s goals and procedures, a return reply form for pastors to indicate their interest in participating, the name(s) of appropriate FBO representative(s) (e.g., leaders of health ministries) to complete the survey, and the preferred mode of contact (phone/email/in-person). Approximately 2 weeks later, bilingual survey assistants called those who provided a return reply (“opt-in”) and attempted to contact those who had not yet responded or opted-out. Prior to participating, organizational consent was obtained from pastors. When the survey respondent was not the pastor, they too provided informed consent.

### Data collection

Surveys were administered by phone, in-person or online, based on respondent preference by trained, bilingual survey assistants. Survey administration took between 20–45 min to complete. Throughout the process of recruitment, we continuously made efforts to verify contact information and addresses, although we were not successful in many circumstances possibly due to closings, reconfigurations, or moves/changes in address, which are all frequent occurrences in FBOs [12]. Data collection took place from 2014 to 2015.

### Measures

Our selection of relevant variables was guided by the Consolidated Framework for Implementation Research (CFIR) [13] as it is among the most widely used frameworks in the field of implementation science [14] and has a robust focus on organizational context. The CFIR describes myriad factors that impact the implementation of innovations in organizational settings, including internal organizational characteristics (“inner setting”) and factors external to the organization (e.g., external policies, incentives). Here, we focus on the inner organizational setting. The CFIR suggests that organizations with access to knowledge, skills, and resources necessary for the implementation of the innovation (“*organizational readiness*”), those that have a collective receptivity to change (positive “*implementation climate*”), and those that have environments of trust, flexibility, and participative decision-making (“*organizational culture*”) are more likely to implement innovations. We also assessed “*innovation-values fit*” (organizational values that are consistent with the innovation) [15], as this has been found important in our prior studies in FBOs [16]. *Structural factors*, (e.g., organizational size), *resources* for health programming (e.g., health ministries), and collaborations with agencies or organizations that address health have also been found important considerations, so they were included here, as well.

Previously, we (JA) conducted a systematic review of measures to assess inner setting organizational characteristics associated with implementation and found no validated measures appropriate to assess latent CFIR constructs among FBOS [17]. Therefore, we adapted existing instruments that had been used in other settings, such as healthcare organizations and schools. Adaptations involved changing terminology so that questions addressed FBOs (e.g., “Your organization [replaced with church] is expected to carry out health programs”).

Measures are described below and sample questions for each construct are presented in Table 1. *Innovation-values fit*, or the perception that these types of health programming fit with the organization’s overall mission and would foster fulfillment of its values, was assessed with 5 items adapted from Belkhodja et al. [18]. *Implementation climate*, or the extent to which the policies and practices of the organization foster, support, and reward program implementation, was gauged with 7 items from Weiner et al. [19]. O*rganizational culture*, which includes organizational norms and values about the implementation of innovations, was assessed with 7 items from Helfrich et al. [20]. *Organizational readiness*, or the shared resolve among individuals within the organization to implement these types of program activities and the collective capacity to do so, was measured with 12 items based on the work of Weiner et al. [19]. For each of the organizational-level constructs above, respondents were asked the extent to which they agreed with statements on a 5-point Likert scale (1 = low agreement, 5 = high agreement). Items were summed for each construct and divided by the total number of items in the scale, with 1 indicating the lowest level and 5 indicating the highest level. We have found these measures to have acceptable internal reliability in our prior FBO studies (alpha > 0.70) [21] and found them to have good internal reliability in this sample (see Table 1).

**Table 1:** CFIR “inner-setting” construct and sample survey questions

Construct	Definition^**a**^	Sample questions
**Structural characteristics**^a^	The social architecture, age, maturity, and size of an organization.	“How many adults attend church services in this church in a typical week?” “How many paid staff are employed by the church?” “How many individuals volunteer on a regular basis?”
**Inner-setting organizational characteristics**		
Innovations and values fit	Perception that these types of health programming fit with the organization’s overall mission and would foster fulfillment of its values.	“Offering health-related activities and programs is relevant to the mission of the church.”
Implementation climate^a^	The absorptive capacity for change, shared receptivity of involved individuals to an intervention, and the extent to which use of that intervention will be 'rewarded, supported, and expected within their organization.'	“Your church is expected to have health-related activities and programs.”
Organizational culture^a^	Norms, values, and basic assumptions of a given organization.	“Church leadership rewards innovation and creativity to improve health programs.”
Organizational readiness^a^	Tangible and immediate indicators of organizational commitment to its decision to implement an intervention, consisting of three subconstructs (leadership engagement, available resources, and access to information and knowledge).	“How confident are you that your church can carry out program activities?” and “How confident are you that your church could find someone who has the interest, skills & time to lead program activities?”
**Resources for health programing**	Existence of persons, committees, or collaborations with other agencies for the purpose of conducting health activities.	“Does your church have any organized committee, effort, designated person, or ministry whose purpose is to coordinate health activities or programs?”

^a^Definitions taken from Damschroder [13]

To assess health programming (our primary outcome), we asked “Has your church participated in or supported health-related projects or programs of any sort to serve the members of your church within the past twelve months?” (yes, no, don’t know) and subsequently, “What type of health-related projects or programs has [church name] sponsored or participated in within the last 12 months?” We characterized health programs as “health education” if the sole purpose was to provide information about health topics or as “health service” programs if they involved the direct provision of health services or health promotion activities (e.g., blood pressure checks).

For *structural characteristics*, we assessed congregation size and leadership characteristics (e.g., number of staff, educational level of pastor). *Resources for health programing* included questions about the existence of health ministries or committees (defined as groups whose mission was to conduct health promotion activities for the congregation). We also inquired about the percentage of the congregation that was actively involved in *volunteer work*, as this may be a resource for delivering programming. *Existing collaborations* with agencies or organizations that could facilitate the implementation of health programs were also assessed.

## Analysis

Our analytic goal was to describe the inner-setting organizational characteristics of FBOs. For all variables, responses of “don’t know” or “refused” were coded as missing. Percent missing data were calculated for each variable. Cases (FBOs) with missing values for the latent organizational constructs of interest were excluded from analysis (*n* = 1). Cronbach’s alpha and composite reliability (CR) were used to measure internal reliability and strength of consistency among items used to assess latent inner-setting organizational constructs (i.e., innovation/values fit, implementation climate, organizational culture, organizational readiness). Composite reliability is a measure of the overall reliability of a collection of distinct but similar items used to create a latent construct. Composite reliability was used to confirm the Cronbach’s alphas in the present study, as it is considered more robust than Cronbach’s alpha, which may be influenced by skewed data. Measures with a Cronbach alpha or a CR of greater than 0.70 were considered to have high inter-reliability [22].

We then conducted a descriptive analysis, including means, standard deviations, medians, and interquartile ranges for continuous variables. Categorical variables were examined with frequencies. As a secondary analytic goal, we assessed associations between organizational characteristics with health programming. We first confirmed that the data met the assumption of equal variance. We then compared mean responses to the organizational characteristics with health programming (yes/no) using Levine’s test [23]. *T* tests were also used to assess whether prior health programming differed significantly by structural characteristics of FBOs. Significance was determined by a *p* value of 0.05 or less. Analyses were done using SAS 9.4 software (Cary, NC) and R version 3.6.3 (R Core Team, 2020) using the *psych* (v1.9.12) [24] and *dplyr* (v 0.8.5) [25] packages.

## Results

### Characteristics of the sample (Table 2)

A total of 140 FBOs were identified as potentially eligible, but 12 had their phones disconnected or there was no church at the identified address, so they were subsequently deemed ineligible. Of the remaining 128, 6 FBOs opted out, 88 never responded to phone or email, and 1 was dropped due to missing data, leaving a final analytic sample of *n* = 34 (34/128 = 26.5%).

**Table 2:** Characteristics of FBOs (*n* = 34)

	Mean or %	SD	Range
**Structural characteristics**			
Number of congregants	121.3	111.0	10–500
% Latino	56.10%	24.8	0–100
Years of Spanish services offered	15.60	14.9	1–70
Number of full-time paid pastoral staff	1.40	2.8	0–15
Number of full-time non-pastoral staff	0.30	0.7	0–2
% pastors with graduate degree	28%		
**Resources for health programming**			
% FBOs with a health ministry	15.0%		
% of members who volunteer	10.8%	11.8	0–40
% FBOs with existing collaborations	3.0%		
**Health programming offerings**			
% of FBOs with health programs (*n* = 7)	20.6%		
Health education:	44.0%		
Health services	33.0%		
Other (e.g., support groups)	11.0%		

In terms of structural characteristics, participating congregations ranged in size from 10 to 500 members (mean = 121, SD = 111). The estimated percentage of the congregation that was Latino/Hispanic varied (15–100%), as did the length of time that a Spanish or bilingual service had been offered (range 1–70, mean = 16 years, SD = 15 years). On average, FBOs had 1.4 full-time paid pastoral staff. FBOs in the sample included a wide variety of Christian-based denominations including Pentecostal (24%), Baptist or Southern Baptist (19%), and Episcopal (13%). The remaining FBOs reported their denominations as Movement of the Living God, Assemblies of God, 7th Day Adventist, Evangelical, First Church of God, or more broadly as “Christian.”

### Inner-setting organizational characteristics (Table 3)

**Fig. 1:**
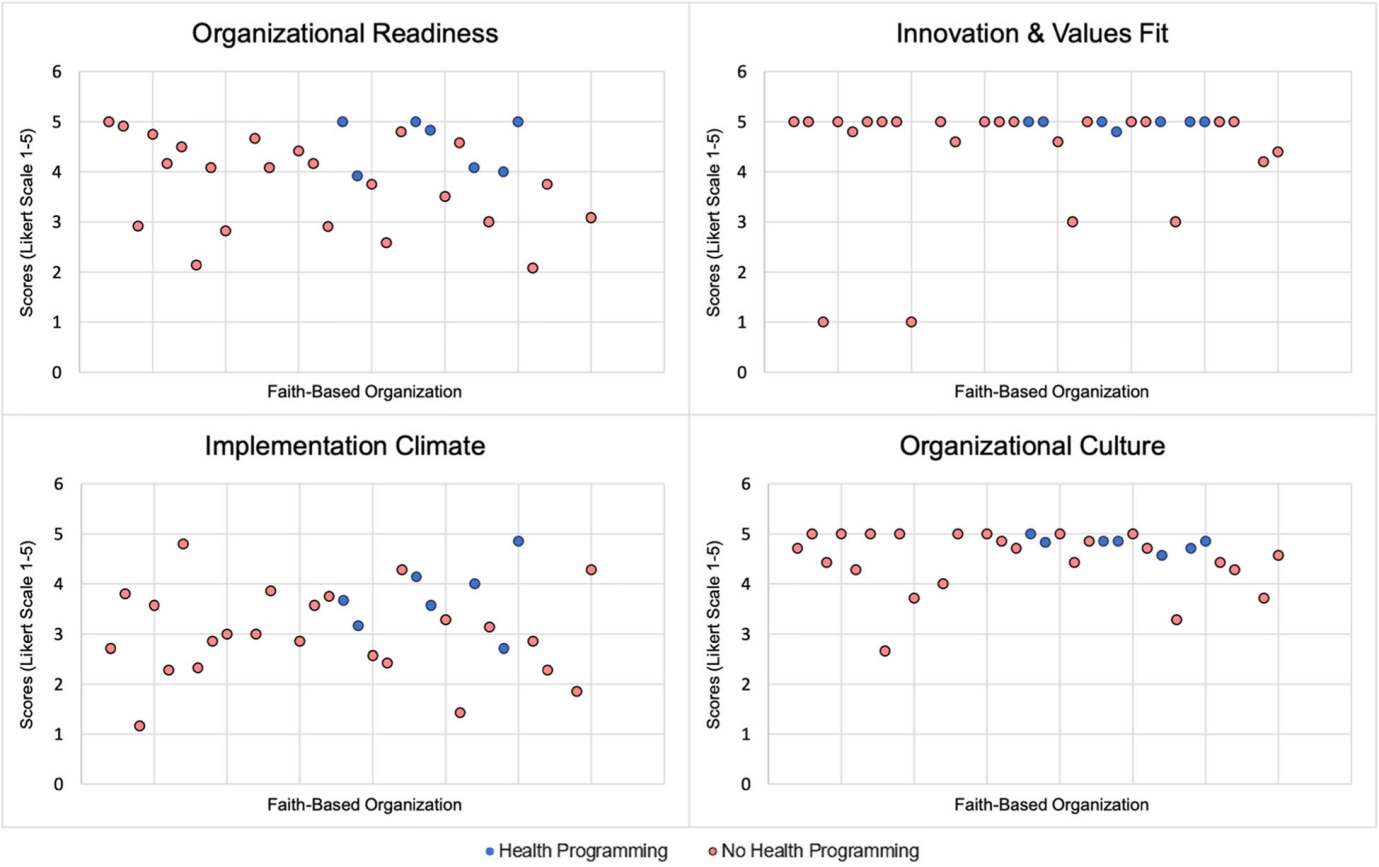
Scatterplots depicting CFIR organizational characteristics across FBOs

All four CFIR characteristics produced both Cronbach’s alpha and composite reliability (CR) scores of greater than 0.70, suggesting that individual items within each construct represent the same organizational construct (Table 2). Across the sample, mean scores on organizational characteristics were high (theoretical range 1–5). The mean score on organizational readiness was 3.86 (SD = 0.92). Perceptions about the innovation-values fit were very high, with a mean score of 4.56 (SD = 1.03). Organizational culture was also high with a mean of 4.58 (SD = 0.54). The score for implementation climate was the lowest across constructs, with a mean of 3.27 (SD = 0.94). Overall, 85% of respondents strongly agreed with the statement that they wanted to offer health-related activities for their congregations. Figure 1 presents scatterplots for organizational constructs. While plots for organizational readiness (top left) and implementation climate (bottom left) display some variation, plots for innovation and values fit (top right) and organizational culture (bottom right) show that scores were highly skewed toward higher values.

**Table 3:** Inner-setting organizational characteristics of FBOs (*n* = 34)

Organizational characteristics	Mean	SD	Median	Composite reliability	Cronbach coefficient alpha
Innovation and values fit	4.56	1.03	5.00	0.76	0.74
Implementation climate	3.27	0.94	3.23	0.74	0.74
Organizational culture	4.58	0.54	4.71	0.97	0.97
Organizational readiness	3.86	0.92	4.08	0.94	0.93

Response categories: 1 = low through 5 = high

### Health programming and inner-setting organizational characteristics (Table 4)

Twenty-one percent of FBOs offered health programming in the prior year. Among churches that offered health programs, the mean number of programs offered was 4.3 (SD = 7.7). About half of the churches (*n* = 4) that offered health programming included *health education*, with the majority focused on nutrition and diabetes. Roughly a third (*n* = 3) involved provision of direct *health services* (e.g., blood pressure) or activities (e.g., Zumba, kickboxing). Of those that reported having offered health programs (*n* = 21%), only 15% of parishes reported having a health ministry (*n* = 5). The percentage of the congregation that volunteered ranged from 0 to 40% (mean 10.8%, SD 11.8). Nearly a third (32%) reported having existing collaborations with hospitals and/or health centers for the purpose of increasing access to services or health information for their congregations.

**Table 4:** Comparison of organizational characteristics of FBOs that offered health programming compared with FBOs that did not

	FBOs with health programming	FBOs without health programming	***p*** value
	Mean (SD)	Mean (SD)	
Innovation and values fit	4.97* (0.08)	4.40 (1.19)	0.04
Implementation climate	3.73 (0.69)	3.00 (0.90)	0.03
Organizational culture	4.81* (0.14)	4.49 (0.62)	0.18
Organizational readiness	4.55* (0.52)	3.77 (0.91)	0.02

*p < 0.05

FBOs that offered health programming had significantly higher mean scores on organizational readiness (4.55 vs 3.77; *p* = 0.04), innovation-values fit (4.97 vs 4.40, *p* = 0.03), and organizational culture (4.81 vs 4.49, *p* = 0.02). Differences in mean scores on measures of implementation climate (3.73 vs. 3.0) were not statistically significant between the two groups (those having offered health programs vs not).

## Discussion

Results from this study suggest that FBOs have many of the inner-setting organizational characteristics thought to be important for the implementation of new health programs. Most respondents strongly endorsed the idea of offering of health programs and activities for their congregations, although only about a fifth had offered them in the prior year. FBOs had uniformly high scores of CFIR inner setting organizational characteristics considered to be important for the implementation of new programs. Moreover, FBOs that offered health programs had significantly higher scores on innovation and values fit, organizational culture, and organizational readiness, although reciprocal causation cannot be ruled out.

Our findings are consistent with a number of other studies that found high levels of interest in offering health programs among FBOs [26, 27]. Our finding that FBOs score highly on measures of organizational readiness suggests that they may be ready organizational partners for adoption of health interventions. In our prior work in the CRUZA study [16], we found that by equipping FBOs with an easy-to-follow implementation guide for evidence-based interventions and providing materials that had been adapted for the cultural and linguistic characteristics of congregations, there was an impressive level of uptake. Of the 31 participating FBOs, all implemented some type of evidence-based intervention for cancer control over a 3-month intervention period, including those in the comparison arm that received only a single phone consultation with an intervention specialist. However, Tagai and colleagues reported that African American churches participating in a health promotion trial varied considerably in their organizational capacity to implement health programs, suggesting that it may be useful to assess implementation-related organizational characteristics prior to partnering to conduct health programs [28].

Our findings additionally suggest that FBOs view health programming as being highly aligned with their own missions and values. Many FBOs already address illness, death, and dying within their congregations. It would not be difficult to frame health promotion programs to congregants and church leaders as important strategies for preventing those outcomes. Such tailoring to context may make implementation more appealing to FBOs and could improve the fit between the setting and intervention.

The organizational culture of FBOs may also be conducive to implementing health programs. There is ample literature documenting the importance of leadership engagement with and support from pastors/church leaders for the successful adoption and implementation of new programs [29]. Leaders can inspire problem-solving and action, and dynamic leaders can create a “shared vision” for the organization [30, 31].

In terms of available resources to support health programming, we found that only 15% of the FBOs had an existing health ministry or committee, a factor found to be associated with implementation of health initiatives. We observed a similar distribution of health ministries (18%) in the CRUZA study among Latino Catholic churches [32]. Studies among African American churches have generally found a higher prevalence of health ministries, with several finding that up to two-thirds had established health ministries [2, 33]. Given the large potential role for health ministries, interventions to establish them in Latino FBOs may facilitate initiation of health programs.

Partnerships between FBOs and health or social service organizations can also be conducive to health programming since they can provide specialized expertise that may be needed. We found that one-third of FBOs already had these relationships, which can be leveraged for health programming. A recent study among African American churches found that 65% had existing collaborations with health clinics or other organizations [34]. This suggests that churches may be adept at forming these relationships, although there may be room to further expand upon faith-based collaborations with health organizations.

Before discussing implications, we acknowledge study limitations. Participation in the survey was suboptimal and since we were unable to directly contact many FBOs, the response rate may not be accurate. Therefore, caution must be used when generalizing these findings. If the FBOs that elected to participate were more interested in health than those that did not respond, this could lead us to overestimate scores on organizational characteristics thought to be conducive to health programming. The sample size in our study also did not allow us to stratify by potentially important factors, such as denomination, geographic location, or types of programs offered. Additionally, the cross-sectional nature of the study does not allow us to conclude whether favorable organizational characteristics led FBOs to implement health programs or vice versa. It is possible that perceptions about inner-setting organizational characteristics became more positive *after* health programs were implemented. Another limitation is the potential measurement error. There were no available validated measures for organizational constructs in FBOs, so our measures are adapted for different types of organizations (e.g., worksites, healthcare). We observed limited variability in scores of inner setting organizational characteristics, which points to the need for validated measures. In the time since this study was conducted, a validated instrument to assess inner organizational setting constructs has been developed [35]. However, this measure was specific to health centers so additional testing is still needed to determine if it would be appropriate for FBO settings. Due to limited variability in scores, our findings also suggest that factors external to the organizations may be more impactful in the adoption and implementation of health programs.

Despite these limitations, our results can help to advance public health initiatives that partner with FBOs. It is one of only a few studies that have examined CFIR organizational characteristics in this setting. While there has been a growing literature focused on African American churches, less attention has been given to understanding adoption and among Latino-serving FBOs. Given the promise of partnering with FBOs to reach underserved Latino populations, it is vital to advance our understanding of how these organizations operate, how best to work with their strengths, and to provide support in needed areas that can facilitate successful implementation initiatives.

If our finding that FBOs offering health programming have more favorable inner-setting organizational characteristics is borne out in prospective studies, results may be useful for practitioners in a number of ways. Understanding organizational characteristics can aid in the identification or selection of FBOs that are primed and ready to engage with health program implementation. As more work in health promotion is conducted in FBOs, it may be important to train practitioners to assess organizational needs and readiness as part of the planning process. A more in-depth understanding of organizational characteristics and capacity among FBOs could additionally aid in the selection of interventions to be implemented. When deciding upon which interventions to implement, effort should be made to ensure a good “fit” between needed and existing capacity for implementation efforts. For example, in FBOs with limited capacity, selection of interventions that require less effort, resources, and specialized expertise might be warranted. Additionally, knowing the strengths and areas of need in FBOs could aid in the development of capacity-building interventions at the organizational level.

This study also points to several important areas for future research. A larger sample of FBOs would allow for more sophisticated analyses, enabling us to examine other potentially important factors, including denomination. Prospective analyses are needed to determine whether favorable organizational characteristics are responsible for, or are a result of, implementation of health programs. Further exploratory work is needed to examine the relative salience of organizational factors in different contexts and to examine their singular, cumulative and potentially synergistic effects. The limited variability we observed in measures of inner-setting organizational characteristics underscores the importance of evaluating other CFIR factors important to the implementation process, including characteristics of the intervention (e.g., complexity) and “outer setting” characteristics (e.g., external policies). As noted previously, there is also a need for further development of measures to assess latent constructs of the CFIR both in terms of validity and sensitivity to change [36].

## Conclusion

Widespread implementation of EBIs to maximize population health requires the engagement of partners within and beyond traditional public health and healthcare settings. FBOs have a long and meaningful record of partnering to deliver health programs for their congregations. Prior initiatives have had success with changing individual behaviors, but challenges remain with regard to establishing organizational infrastructures/capacity to sustain programming [37]. Our findings suggest that FBOs have many of the inner-organizational characteristics specified by CFIR to facilitate implementation. A greater understanding of these characteristics—and how best to harness organizational resources and capacity—could advance efforts to disseminate and scale EBIs for broader implementation in a greater number of FBOs and across a variety of audiences.

## Data Availability

If requested, that data may be shared at the discretion of the principal investigator, with requests considered on a case-by-case basis and with an executed data use agreement in place

## Abbreviations

FBOs: Faith-based organizations
EBI: Evidence-based intervention
CFIR: Consolidated Framework for Implementation Research
